# Validation of genomic predictions for body weight in broilers using crossbred information and considering breed-of-origin of alleles

**DOI:** 10.1186/s12711-019-0481-7

**Published:** 2019-07-08

**Authors:** Pascal Duenk, Mario P. L. Calus, Yvonne C. J. Wientjes, Vivian P. Breen, John M. Henshall, Rachel Hawken, Piter Bijma

**Affiliations:** 1 Animal Breeding and Genomics, Wageningen University and Research, P.O. Box 338, 6700 AH Wageningen, The Netherlands; 2 Cobb-vantress Inc., Siloam Springs, AR 72761-1030 USA

## Abstract

**Background:**

Pig and poultry breeding programs aim at improving crossbred (CB) performance. Selection response may be suboptimal if only purebred (PB) performance is used to compute genomic estimated breeding values (GEBV) because the genetic correlation between PB and CB performance ($r_{pc}$) is often lower than 1. Thus, it may be beneficial to use information on both PB and CB performance. In addition, the accuracy of GEBV of PB animals for CB performance may improve when the breed-of-origin of alleles (BOA) is considered in the genomic relationship matrix (GRM). Thus, our aim was to compare scenarios where GEBV are computed and validated by using (1) either CB offspring averages or individual CB records for validation, (2) either a PB or CB reference population, and (3) a GRM that either accounts for or ignores BOA in the CB individuals. For this purpose, we used data on body weight measured at around 7 (BW7) or 35 (BW35) days in PB and CB broiler chickens and evaluated the accuracy of GEBV based on the correlation GEBV with phenotypes in the validation population (validation correlation).

**Results:**

With validation on CB offspring averages, the validation correlation of GEBV of PB animals for CB performance was lower with a CB reference population than with a PB reference population for BW35 ($r_{pc}$ = 0.96), and about equal for BW7 ($r_{pc}$ = 0.80) when BOA was ignored. However, with validation on individual CB records, the validation correlation was higher with a CB reference population for both traits. The use of a GRM that took BOA into account increased the validation correlation for BW7 but reduced it for BW35.

**Conclusions:**

We argue that the benefit of using a CB reference population for genomic prediction of PB animals for CB performance should be assessed either by validation on CB offspring averages, or by validation on individual CB records while using a GRM that accounts for BOA in the CB individuals. With this recommendation in mind, our results show that the accuracy of GEBV of PB animals for CB performance was equal to or higher with a CB reference population than with a PB reference population for a trait with an $r_{pc}$ of 0.8, but lower for a trait with an $r_{pc}$ of 0.96. In addition, taking BOA into account was beneficial for a trait with an $r_{pc}$ of 0.8 but not for a trait with an $r_{pc}$ of 0.96.

**Electronic supplementary material:**

The online version of this article (10.1186/s12711-019-0481-7) contains supplementary material, which is available to authorized users.

## Background

In pig and poultry breeding programs, purebred (PB) animals from different lines or breeds are mated to produce crossbred (CB) production animals. Although the aim of such breeding programs is to improve CB performance, typically, breeding values of PB selection candidates are estimated using only information on PB performance. As a result, response to selection in CB performance may be suboptimal because the genetic correlation between PB and CB performance ($r_{pc}$) is often lower than 1 [1–3]. A low $r_{pc}$ may be due to genotype-by-environment interactions [4, 5], genotype-by-genotype interactions (i.e., dominance and epistasis) in combination with differences in allele frequencies between the purebred parental lines [6], and/or differences in the definition of PB and CB performance traits [7, 8].

When the $r_{pc}$ is lower than 1, it may be beneficial to use information on both PB and CB performance to estimate breeding values of PB selection candidates. For this strategy, breeders need to be able to connect observations on CB performance to the PB selection candidates. This connection can be established with a pedigree-based relationship matrix. However, in a CB breeding scheme, breeders do not always routinely record pedigree information. In such cases, the pedigree-based relationship matrix can be replaced by a genomic relationship matrix (GRM) that is based on observed marker genotypes [9]. This GRM enables breeders to use a reference population that consists of animals with phenotypes and genotypes to estimate genomic estimated breeding values (GEBV) of selection candidates that only have records on genotypes [10]. When pedigree information is available, replacing the pedigree-based relationship matrix by a GRM may increase the accuracy of estimated breeding values [11]. As such, this method, called genomic prediction, allows breeders to use a CB reference population to compute GEBV for CB performance of PB selection candidates [4].

Simulation studies have suggested that a CB reference population may yield more accurate GEBV for CB performance than a PB reference population when the $r_{pc}$ is lower than 0.8 [4, 12, 13]. This result was shown for situations for which the CB reference population had at least the same size as the alternative PB reference population and the selection candidates had similar relationships to the CB and the PB reference populations. In agreement with these simulation studies, Hidalgo et al. [14], using real data in pigs, found that for a trait with a high $r_{pc}$ (~ 0.90), the accuracy of GEBV of PB animals for CB performance was lower with a reference population of CB compared to PB pigs. These results were not only due to a high $r_{pc}$, but also to the smaller number of CB pigs compared to PB pigs in the reference population, and weaker relationships of the PB selection candidates with the CB reference population than with the PB reference population [14]. In summary, the expected benefit of using a CB reference population instead of a PB reference population increases with (1) lower $r_{pc}$, (2) stronger relationships of the CB reference population with PB selection candidates, and (3) larger sizes of the CB reference population.

When a CB reference population is used to estimate GEBV of PB selection candidates, relationships in the GRM (i.e. ${\mathbf{G}}$) are often constructed while ignoring the breed-of-origin of alleles (BOA) of the CB animals. Thus, one assumes that the apparent effects of markers are the same for alleles that originate from the sire breed and the dam breed. Thus, apparent effects of markers are assumed to be equal across breeds, which may not be valid because of differences in linkage disequilibrium (LD), and/or in allele frequencies between the parental breeds [15–19]. In addition, actual effects at causal loci may differ between breeds due to genotype-by-environment interactions [4, 5] and/or the presence of non-additive effects in combination with differences in allele frequencies [20, 21]. Thus, considering BOA when constructing the GRM may lead to more accurate GEBV.

Recently, a method has been developed that allows the BOA in CB animals to be determined based on phased genotypes, while taking advantage of the known crossbreeding structure [22]. This allows the construction of a partial genomic relationship matrix (${\mathbf{G}}_{BOA}$) [23, 24], in which relationships that involve CB animals are based only on alleles that originate from the line of selection candidates for which GEBV are estimated. Simulation studies suggested that genomic prediction models that take BOA into account may outperform models that ignore it [13, 23]. However, this benefit of considering BOA was only observed when the CB reference population was large (4000), the number of markers was small (500), and the parental lines of CB animals were distantly related. Moreover, empirical studies on pigs suggested that taking BOA into account may increase the accuracy of GEBV only when $r_{pc}$ and heritability are low [25, 26].

In summary, the use of CB information instead of PB information and taking BOA into account may be beneficial for genomic evaluation of PB animals for CB performance. Such benefits are expected when $r_{pc}$ is low but, to date, this hypothesis has not been tested in broiler breeding programs. Furthermore, it is not yet clear how such benefits should be evaluated, i.e. how GEBV from such models should be validated. Thus, the aim of our study was to compare scenarios in which GEBV of PB animals for CB performance are computed and validated by using (1) either CB offspring averages or individual CB records for validation, (2) either a PB or CB reference population, and (3) a GRM that either accounts for or ignores BOA in the CB individuals. Scenarios were compared based on the correlation of GEBV with validation records (hereafter called the validation correlation) and based on the regression coefficient of validation records on GEBV (i.e. bias). For this purpose, we used data on body weight measured at around 7 (BW7) or 35 days (BW35) of age in PB and CB broilers.

## Methods

Previously, in Duenk et al. [27], we estimated genetic parameters for BW7 and BW35 with data from PB and CB animals that were housed in the same environment and that originated from a common group of sires. The estimated heritability of BW7 was 0.09 for PB performance and 0.18 for CB performance, and that of BW35 was 0.22 for PB performance and 0.23 for CB performance. The estimates of $r_{pc}$ for BW7 and BW35 were 0.80 and 0.96, respectively. Furthermore, for the CB animals in this dataset, BOA were derived by Calus et al. [28], which allowed us to consider BOA for genomic prediction. In the current study, we will use the same data as from the previous study to estimate GEBV of PB animals for CB performance, with a reference population of either PB or CB animals, and validate those GEBV with either CB offspring averages or individual CB records.

We used phenotype data on body weight from male and female offspring from a PB sire line (A), and from a three-way crossbred population (A(BC)). The three-way crossbred offspring resulted from mating sires of line A with F1 dams that were a cross between dam lines B and C (BC). All PB and CB offspring came from the same generation and were generated using the same PB line A sires in order to create sufficient links between the PB and CB offspring to enable accurate estimation of $r_{pc}$. The dam lines used (B and C) have been selected on egg production traits, whereas the sire line A has been selected on male fertility traits, along with growth, yield, and feed efficiency. The three parent lines (A, B, C) were genetically distant, as shown by the principal component analysis in Duenk et al. [27].

Our aim was to investigate the effect of the validation records used (CB offspring averages or CB individual records) on the validation correlation and bias based on linear regression of validation records on GEBV. Our first strategy was to validate PB sire GEBV for CB performance with CB offspring averages (scenarios –A, Table 1). However, because the number of sires was small (161), we expected a relatively large standard error of the resulting validation correlation. Thus, our second strategy was to validate GEBV for CB performance with individual CB records (scenarios –I, Table 1), following Xiang et al. [29]. For both these validation methods, we compared the validation correlation and bias for GEBV obtained using either a PB reference population (scenarios PB-A and PB-I, Table 1) or a CB reference population (scenarios CB-A and CB-I, Table 1). With a CB reference population, we also investigated the benefit of considering the BOA (CB-A-BOA and CB-I-BOA, Table 1). Note that, in this study, we did not use own performance records of the purebred selection candidates, because we wanted to compare the predictive value of a CB reference population with that of PB reference population, both consisting of animals that are not closely related to the selection candidates.

**Table 1:** Overview of scenarios with information on the types of reference population, validation records, and genomic relationship matrix (GRM) that were used

Scenario^a^	Reference population	Prediction	Validation	GRM
PB-A	PB offspring	Sire GEBV	Offspring averages	${\mathbf{G}}$
CB-A	CB offspring	Sire GEBV	Offspring averages	${\mathbf{G}}$
CB-A-BOA	CB offspring	Sire GEBV	Offspring averages	${\mathbf{G}}_{BOA}$
PB-I	PB offspring	CB GEBV	Individual records	${\mathbf{G}}$
CB-I	CB offspring	CB GEBV	Individual records	${\mathbf{G}}$
CB-I-BOA	CB offspring	CB GEBV	Individual records	${\mathbf{G}}_{BOA}$

^a^In the abbreviation of the scenarios, the first element indicates the reference population (PB or CB), the second element the validation record (CB offspring averages indicated by A or individual offspring records indicated by I), and a third element “BOA” is added for scenarios that consider BOA

### Phenotype data

For recording phenotype data, a single generation of offspring were weighed at around 7 (BW7) and 35 (BW35) days of age in five consecutive batches of similar size, with both PB and CB offspring in every batch. The five batches followed each other directly, and together spanned less than five months. Birds from the first batch hatched in June 2014, and those from the last batch hatched in November 2014. Animals that belonged to the offspring generation in one of the batches were not parents of birds in any of the other batches. Within each batch, the PB and CB offspring were housed in three to five pens. For 16 out of 20 pen-batch combinations, at least 90% of the animals in the pen were from the same genetic group (i.e. PB or CB animals), while for the remaining pens, between 53 and 77% of the animals in the pen were from the same genetic group. Each pen had a near equal number of males and females. Each sire had most of its offspring housed in the same pen, and each pen had offspring of multiple sires. Outlier analysis was done separately per day of recording, and separately for PB and CB animals. Observations that deviated more than 3.5 standard deviations from the mean were removed. After removing the outliers, 4687 PB and 10,585 CB records remained for BW7, and 4471 PB and 10,272 CB records remained for BW35. The number of animals with observations was smaller for BW35 than for BW7, because some animals did not survive until 35 days.

### Genotype data

Genotypes were collected from all PB and CB offspring with phenotypes, as well as from their potential parents and from most of their potential grandparents. Marker positions were determined based on the *Gallus gallus* 4.0 (galGal4) reference assembly. Genotype markers were removed if they were located on sex chromosomes or on the mitochondrial genome, had unknown locations, or a call rate lower than 90%. Animals were removed from the genotype data if they had a call rate lower than 90%. The remaining genotypes were used to reconstruct the pedigree, so that pedigree information was available up to the generation of the grandparents. Genotypes of the grandparents were only used to assign BOA for the animals with phenotypes. In total, there were 161 unique PB sires from line A, of which 135 sires had both PB and CB offspring, five sires had only PB offspring, and 21 sires had only CB offspring (Table 2). The PB offspring had 628 unique dams, whereas the CB offspring had 1028 unique dams.

**Table 2:** Summary statistics for body weight measured around 7 (BW7) and around 35 days of age (BW35)

		Number	Number of sires	Number of dams	Mean (g)	sd (g)
BW7	Purebreds	4687	142	628	176	25
	Crossbreds	10,585	156	1028	179	23
	Total	15,272	161^a^	1656		
BW35	Purebreds	4471	140	623	2066	303
	Crossbreds	10,272	156	1027	2090	302
	Total	14,743	161^a^	1650		

^a^Total number of sires for all purebred and crossbred animals

We used the reconstructed pedigree to check the genotypes of each marker for Mendelian inheritance inconsistencies between all parent–offspring pairs. Markers with more than 1% inconsistent genotypes between parent–offspring pairs were removed, and for the remaining identified inconsistencies, the genotypes of parent and offspring were set to missing. No animal had more than 1% of inconsistencies across markers. All missing genotypes were imputed with FImpute [30]. After assigning BOA, we removed markers if they had a minor allele frequency lower than 0.005 in either the genotype file or the BOA file. After these edits, 50,960 markers remained for analysis.

### Assigning breed-of-origin of alleles

For all markers, the BOA in the CB offspring were derived with the BOA approach [22, 31]. In short, the BOA approach consists of (1) simultaneously phasing genotypes of PB and CB animals with AlphaPhase 1.1 using pedigree information [32], (2) collecting a library of haplotypes for each line using these phased haplotypes, and (3) assigning the BOA in the CB animals. Steps 2 and 3 were performed using in-house software. This approach resulted in 49.5% of the alleles being assigned to sire line A, which is close to the expected 50%. The full procedure and results of assigning BOA in these data are described in Calus et al. [28].

### Data selection

The available number of CB animals with phenotypes and genotypes was more than twice as large as the number of PB animals (Table 2). However, our aim was to compare the use of a PB reference population to that of a CB reference population of similar size. Thus, we randomly selected a set of ~ 4500 CB animals to be used in the analyses, while aiming for a comparable family structure in the PB data and the selected set of CB animals. To this end, we counted the number of PB full-sib families of size $s$ (ranging from 1 to 11) and we randomly selected the same number of CB full-sib families of size $s$. If the available number of CB families of size $s$ was smaller than the number of PB families of size $s$, all CB families of this size were selected (Table 3). As a result, the number of CB offspring in the selected set was 4655 for BW7 and 4445 for BW35. These numbers were only slightly smaller than the corresponding numbers of PB offspring (4687 for BW7 and 4471 for BW35).

**Table 3:** Number of full-sib families in the PB and CB offspring by family size

Family size	Number of PB families	Number of CB families		Average fraction overlap^a^
		Total	Selected	
1	1699	4406	1699	0.39
2	653	1610	653	0.40
3	276	607	276	0.46
4	117	177	117	0.66
5	46	60	46	0.77
6	13	14	13	0.93
7	6	3	3	1.00
8	2	2	2	1.00
9	1	1	1	1.00
10	0	0	0	–
11	1	0	0	–

^a^The average fraction of CB animals that two randomly selected sets of CB animals (replicates) had in common, computed per family size

An initial analysis revealed that the validation correlation from using a CB reference population differed substantially between randomly selected sets of CB animals. To reduce the impact of this variability on the outcome of the study, we independently sampled 100 different sets of CB animals using the procedure described above. The average fraction of CB animals that two sets had in common for each family size is in Table 3; the overall average fraction was 0.47.

### Genomic prediction and cross-validation populations

We ran all scenarios for each of the 100 sets of ~ 4500 CB animals separately, resulting in 100 replicates for each scenario. For every replicate, we used the selected set of CB animals or all PB animals with phenotypes to create the reference and validation population following the cross-validation strategy explained in the next paragraph. For scenarios denoted by CB-A and CB-A-BOA, the selected CB set was used to create reference populations, and CB offspring averages of sires were used for validation; for scenarios CB-I and CB-I-BOA, the selected CB set was used to create both the reference and validation populations; for scenario PB-I, the selected CB set was used to create the validation populations, and all PB offspring were used to create the reference populations; for scenario PB-A, all PB offspring were used to create the reference populations and CB offspring averages of sires were used for validation.

For each replicate, our aim was to minimise relationships between animals in the reference and animals in the validation population by creating five cross-validation (CV) groups. The CV groups were created so that animals in the validation population did not have offspring or paternal-half sibs in the reference population. Thus, we randomly assigned the 156 PB sires that had CB offspring to these CV groups, such that four groups had 32 sires and one group had 33 sires. All offspring were then assigned to the same CV group as their sire. For each CV group, either the sires (for validation on CB offspring averages) or the CB animals (for validation on individual CB records) in this group were used as the validation population, while either the PB or CB offspring in the remaining CV groups were used as the reference population (Table 1). The PB offspring of sires without CB offspring were always included in the PB reference population.

GEBV were predicted separately for BW7 and BW35 with the following univariate model:

1 $${\mathbf{y}} = {\mathbf{Xb}} + {\mathbf{Lm}} + {\mathbf{Za}} + {\mathbf{e}}\text{,}$$

where ${\mathbf{y}}$ is a vector of phenotypes, ${\mathbf{b}}$ is a vector of fixed effects (batch $\times$ pen $\times$ sex $\times$ age at measurement) with design matrix ${\mathbf{X}}$, ${\mathbf{m}}$ is a vector of permanent environmental (maternal) effects with incidence matrix ${\mathbf{L}}$, ${\mathbf{a}}$ is a vector of additive genetic effects with incidence matrix ${\mathbf{Z}}$, and ${\mathbf{e}}$ is a vector of random residuals. The distribution of permanent environmental (maternal) effects was assumed ${\mathbf{m}} \sim\,N\left( {0,{\mathbf{I}}_{m} \sigma_{m}^{2} } \right)$, where $\sigma_{m}^{2}$ is the permanent environmental variance and ${\mathbf{I}}_{m}$ is an identity matrix. The distribution of additive genetic effects was assumed ${\mathbf{a}} \sim\,N\left( {0,{\mathbf{G}}\sigma_{a}^{2} } \right)$, where $\sigma_{a}^{2}$ is the additive genetic variance and ${\mathbf{G}}$ is a multi-breed genomic relationship matrix that either ignores or considers BOA (${\mathbf{G}}_{BOA}$). The distribution of residuals was assumed **e** $\sim\,N\left( {0,{\mathbf{I}}_{r} \sigma_{e}^{2} } \right)$, where $\sigma_{e}^{2}$ is the residual variance and ${\mathbf{I}}_{r}$ is an identity matrix.

For scenarios CB-A and PB-I, matrix ${\mathbf{G}}$ was constructed following Wientjes et al. [33]:

2 $$\begin{aligned} {\mathbf{G}} & = \left[ {\begin{array}{*{20}c} {{\mathbf{G}}_{\text{PB}} } & {{\mathbf{G}}_{{{\text{PB}} - {\text{CB}}}} } \\ {{\mathbf{G}}_{{{\text{CB}} - {\text{PB}}}} } & {{\mathbf{G}}_{\text{CB}} } \\ \end{array} } \right] \\ & = \left[ {\begin{array}{*{20}c} {\frac{{{\mathbf{M}}_{\text{PB}} {\mathbf{M}}_{\text{PB}}^{ '} }}{{\sum 2p_{j}^{PB} \left( {1 - p_{j}^{PB} } \right)}}} & {\frac{{{\mathbf{M}}_{\text{PB}} {\mathbf{M}}_{\text{CB}}^{\varvec{'}} }}{{\sqrt {\sum 2p_{j}^{PB} \left( {1 - p_{j}^{PB} } \right)} \sqrt {\sum 2p_{j}^{CB} \left( {1 - p_{j}^{CB} } \right)} }}} \\ {\frac{{{\mathbf{M}}_{\text{CB}} {\mathbf{M}}_{\text{PB}}^{\varvec{'}} }}{{\sqrt {\sum 2p_{j}^{PB} \left( {1 - p_{j}^{PB} } \right)} \sqrt {\sum 2p_{j}^{CB} \left( {1 - p_{j}^{CB} } \right)} }}} & {\frac{{{\mathbf{M}}_{\text{CB}} {\mathbf{M}}_{\text{CB}}^{\varvec{'}} }}{{\sum 2p_{j}^{CB} \left( {1 - p_{j}^{CB} } \right)}}} \\ \end{array} } \right], \\ \end{aligned}$$

where ${\mathbf{M}}_{\text{CB}}$ and ${\mathbf{M}}_{\text{PB}}$ are a centred marker genotype matrix of CB animals and PB animals, respectively, by subtracting $2p_{j}^{CB}$ (for ${\mathbf{M}}_{\text{CB}}$) or $2p_{j}^{PB}$ (for ${\mathbf{M}}_{\text{PB}}$) from all genotypes of marker $j$, where $p_{j}^{CB}$ and $p_{j}^{PB}$ are the allele frequency of marker $j$ in the CB and PB animals, respectively. For scenarios PB-A and CB-I, either PB or CB animals were involved, so the ${\mathbf{G}}$ matrix in Eq. (2) reduced to the genomic relationship matrix for a single breed: ${\mathbf{G}} = \frac{{{\mathbf{MM^{\prime}}}}}{{\sum 2p_{j} \left( {1 - p_{j} } \right) }}$ [9].

When BOA was considered and validation was based on offspring averages (CB-A-BOA), the genomic relationships in ${\mathbf{G}}_{BOA}$ were constructed by using only the alleles that came from sire line A as:

3 $$\begin{aligned} {\mathbf{G}}_{BOA} & = \left[ {\begin{array}{*{20}c} {{\mathbf{G}}_{\text{PB}} } & {{\mathbf{G}}_{{{\text{PB}} - {\text{CB}}}} } \\ {{\mathbf{G}}_{{{\text{CB}} - {\text{PB}}}} } & {{\mathbf{G}}_{\text{CB}} } \\ \end{array} } \right] \\ & = \left[ {\begin{array}{*{20}c} {\frac{{{\mathbf{M}}_{\text{PB}} {\mathbf{M}}_{\text{PB}}^{ '} }}{{\sum 2p_{j}^{PB} \left( {1 - p_{j}^{PB} } \right)}}} & {\frac{{{\mathbf{M}}_{\text{PB}} {\mathbf{M}}_{\text{CB}}^{\varvec{'}} }}{{\sqrt {\sum 2p_{j}^{PB} \left( {1 - p_{j}^{PB} } \right)} \sqrt {\sum 2p_{j}^{CB} \left( {1 - p_{j}^{CB} } \right)} }}} \\ {\frac{{{\mathbf{M}}_{\text{CB}} {\mathbf{M}}_{\text{PB}}^{\varvec{'}} }}{{\sqrt {\sum 2p_{j}^{PB} \left( {1 - p_{j}^{PB} } \right)} \sqrt {\sum 2p_{j}^{CB} \left( {1 - p_{j}^{CB} } \right)} }}} & {\frac{{{\mathbf{M}}_{\text{CB}} {\mathbf{M}}_{\text{CB}}^{\varvec{'}} }}{{\sum 2p_{j}^{CB} \left( {1 - p_{j}^{CB} } \right)}}} \\ \end{array} } \right], \\ \end{aligned}$$

where ${\mathbf{T}}_{CB}$ is the centred marker allele matrix for CB animals, with a value of $\left( {1 - p_{j} } \right)$ if the counted allele was inherited from the PB sire line, and a value of $\left( {0 - p_{j} } \right)$ if the other allele was inherited [26], where $p_{j}$ denotes the frequency of the counted allele at marker $j$. The latter was calculated as the total number of counted alleles in the PB sires and in the CB offspring that were inherited from these sires, divided by the total number of PB alleles in these animals. Note that the ${\mathbf{G}}_{BOA}$ is similar to the marker-based partial relationship matrix from Christensen et al. [24], with a scaling factor of $\sum 2p_{j} \left( {1 - p_{j} } \right)$. As a result, the expected value of the diagonal elements for CB animals in ${\mathbf{G}}_{BOA}$ is 0.5. For scenario CB-I-BOA, only CB animals were involved, so ${\mathbf{G}}_{BOA}$ from Eq. (3) reduced to a genomic relationship matrix between CB animals where only alleles from sire line A were considered, i.e. ${\mathbf{G}}_{BOA} = \frac{{{\mathbf{T}}_{{{\mathbf{CB}}}} {\mathbf{T}}_{{{\mathbf{CB}}}}^{'} }}{{\sum 2p_{j} \left( {1 - p_{j} } \right) }}$.

### Validation records, validation correlation and bias

Phenotypic records corrected for systematic environmental effects were used for validation and were obtained from the following model, separately for BW7 and BW35:

4 $${\mathbf{y}} = {\mathbf{Xb}} + {\mathbf{Lm}} + {\mathbf{Ts}} + {\mathbf{e}},$$

where ${\mathbf{y}}$ is a vector of all available CB phenotypes, ${\mathbf{s}}$ is a vector of random sire effects with incidence matrix ${\mathbf{T}}$, and all other terms are the same as in Eq. (1). The distribution of sire effects was assumed ${\mathbf{s}} \sim\,N\left( {0,{\mathbf{I}}_{s} \sigma_{s}^{2} } \right)$, where $\sigma_{s}^{2}$ is the sire variance and ${\mathbf{I}}_{s}$ is an identity matrix. From the solutions of this model, corrected phenotypes were computed as ${\mathbf{y}}_{\text{c}} = {\mathbf{T}}\hat{\mathbf{s}} + {\hat{\mathbf{e}}}$.

The validation correlation and bias were evaluated for each replicate separately, using the GEBV and validation records of validation animals from all CV groups. For validation on individual CB records, the validation correlation was calculated as the correlation between GEBV and corrected individual CB records (${\mathbf{y}}_{\text{c}}$) and the bias was calculated by regressing ${\mathbf{y}}_{\text{c}}$ on GEBV. For validation on CB offspring averages, the validation correlation was calculated as the weighted correlation between the sire GEBV and the average of corrected phenotypes of their CB offspring ($\overline{{{\mathbf{y}}_{\text{c}} }}$) and bias was calculated by weighted regression of $\overline{{{\mathbf{y}}_{\text{c}} }}$ on sire GEBV, with the weighted regression coefficient multiplied by two because the offspring average represents half the breeding value of the sire. The weights used in these analyses were the reliabilities of $\overline{{{\mathbf{y}}_{\text{c}} }}$, which were computed as $\frac{{\frac{1}{4}nh_{CB}^{2} }}{{1 + \frac{1}{4}\left( {n - 1} \right)h_{CB}^{2} }}$ [34], where $h_{CB}^{2}$ is the estimated heritability of CB performance and $n$ is the number of CB offspring. Note that the resulting validation correlations are not equal to but are proportional to the accuracies of the GEBV for a given validation population, defined as the correlation between GEBV and true breeding values in validation. The validation correlations therefore allow for a comparison between scenarios, which was the aim of our study.

## Results

### PB versus CB reference population

For BW7 and with validation on offspring averages, the PB and CB reference populations yielded a similar mean validation correlation (both equal to 0.16; Table 4). With validation on individual CB records, however, the CB reference population yielded a higher mean validation correlation than the PB reference population (0.13 vs. 0.05; Table 4). For BW35 and with validation on CB offspring averages, the CB reference population yielded a lower mean validation correlation than the PB reference population (0.26 vs. 0.36; Table 4). With validation on individual CB records, the CB reference population yielded a higher mean validation correlation than the PB reference population (0.16 vs. 0.13; Table 4).

**Table 4:** Mean validation correlations for BW7 and BW35

Scenario	Reference	Validation	BW7		BW35	
			Mean^a^	sd^b^	Mean^a^	sd^b^
PB-A	PB	Offspring averages	0.16	0.032	*0.36*	0.032
CB-A	CB	Offspring averages	0.16	0.058	0.26	0.060
CB-A-BOA	CB	Offspring averages	*0.20*	0.058	0.22	0.059
PB-I	PB	Individual records	0.05	0.014	0.13	0.014
CB-I	CB	Individual records	*0.13*	0.020	*0.16*	0.020
CB-I-BOA	CB	Individual records	0.08	0.025	0.09	0.025

^a^Reported values are means of 100 replicates. Highest mean validation correlations per validation record and per trait are in italics

^b^Reported values are standard deviations of validation correlations of 100 replicates

**Fig. 1:**
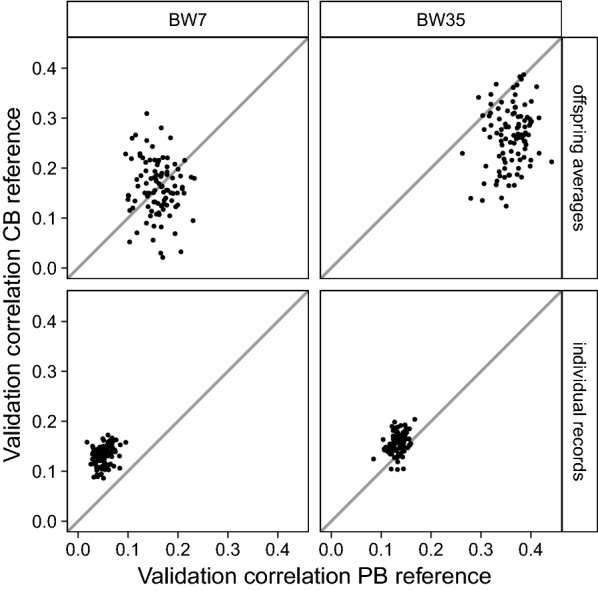
Validation correlations when validation was on CB offspring averages or individual CB records, using a PB or a CB reference population. The x-axis represents the validation correlation using a PB reference population and the y-axis represents the validation correlation using a CB reference population. Panels refer to validation on CB offspring averages or individual CB records across rows, and to body weight measured at around 7 (BW7) or 35 (BW35) days across columns. Dots represent individual validation correlations of 100 replicates and straight lines indicate x = y

The differences between mean validation correlations were not always larger than their standard errors and, thus, we examined if these observed differences were consistent for individual validation correlations of replicates. For BW7 and with validation on CB offspring averages, there was no clear difference between a PB and a CB reference population (Fig. 1, top-left); in 51% of the replicates, the validation correlation was higher for the CB reference population. However, with validation on individual CB records, the validation correlation was higher with a CB reference population for all replicates (Fig. 1, bottom-left). For BW35 and with validation on CB offspring averages, the PB reference population yielded a higher validation correlation than a CB reference population for 93% of the replicates (Fig. 1, top-right). However, with validation on individual CB records the CB reference population mostly yielded a higher validation correlation (86% of the replicates; Fig. 1, bottom-right).

**Fig. 2:**
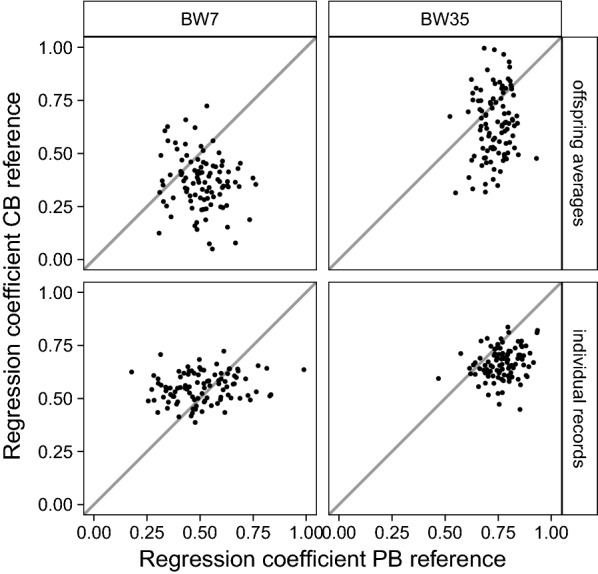
Regression coefficients of validation records on GEBV when validation was on CB offspring averages or individual CB records, using a PB or a CB reference population. The x-axis represents the regression coefficient using a PB reference population and the y-axis represents the regression coefficient using a CB reference population. Panels refer to validation on CB offspring averages or individual CB records across rows, and to body weight measured at around 7 (BW7) or 35 (BW35) days across columns. Dots represent individual regression coefficients of 100 replicates, and straight lines indicate x = y

There were no clear differences in bias of GEBV between using a PB or CB reference population, except for BW35 and with validation on individual offspring records. For that scenario, GEBV from the PB reference population were less biased in 87% of the replicates (Fig. 2, bottom-right), with a mean regression coefficient of 0.77 for the PB reference population and 0.67 for the CB reference population (Table 5).

**Table 5:** Mean regression coefficients of GEBV on validation records for BW7 and BW35

Scenario	Reference	Validation	BW7		BW35	
			Mean^a^	sd^b^	Mean^a^	sd^b^
PB-A	PB	Offspring averages^c^	0.51	0.105	*0.73*	0.069
CB-A	CB	Offspring averages^c^	0.36	0.133	0.64	0.158
CB-A-BOA	CB	Offspring averages^c^	*0.55*	0.171	0.59	0.167
PB-I	PB	Individual records	0.51	0.147	*0.77*	0.080
CB-I	CB	Individual records	0.55	0.070	0.67	0.073
CB-I-BOA	CB	Individual records	*0.67*	0.202	0.64	0.169

^a^Reported values are means of 100 replicates. Mean regression coefficients that are closest to 1 per validation record and per trait are in italics

^b^Reported values are standard deviations of regression coefficients of 100 replicates

^c^Reported regression coefficients were multiplied by 2 because offspring averages represent half the breeding value of sires

### Ignoring versus considering BOA

**Fig. 3:**
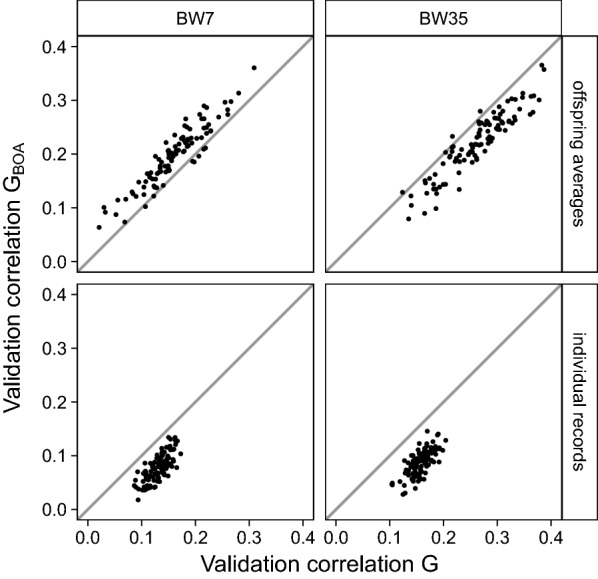
Validation correlations when validation was on CB offspring averages or individual CB records, the reference population consisted of CB animals, and BOA was ignored or considered. The x-axis represents the validation correlation when ignoring BOA and the y-axis represents the validation correlation when considering BOA. Panels refer to validation on CB offspring averages or individual CB records across rows, and to body weight measured at around 7 (BW7) or 35 (BW35) days across columns. Dots represent individual validation correlations of 100 replicates, and straight lines indicate x = y

With validation on offspring averages, considering BOA increased the mean validation correlation for BW7 (0.20 vs. 0.16; Table 4), but decreased the mean validation correlation for BW35 (0.22 vs. 0.26; Table 4). With validation on individual CB records, considering BOA decreased the mean validation correlation for both BW7 (0.08 vs. 0.13; Table 4) and BW35 (0.09 vs. 0.16; Table 4). Again, we examined whether the observed differences in mean validation correlations were consistent for individual replicates. With validation on CB offspring averages, taking BOA into account almost always increased the validation correlation for BW7 (93% of the replicates; Fig. 3, top-left), whereas for BW35, it almost never increased it (3% of the replicates; Fig. 3, top-right). With validation on individual CB records, taking BOA into account never increased the validation correlation for either BW7 or BW35 (Fig. 3, bottom).

**Fig. 4:**
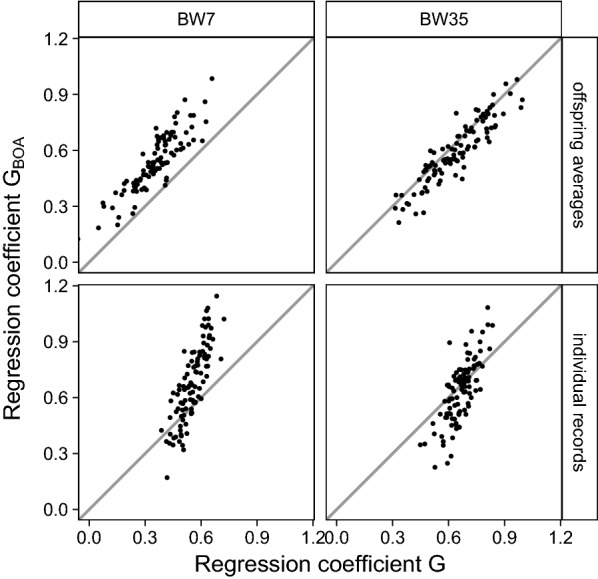
Regression coefficients of validation records on GEBV when validation was on CB offspring averages or individual CB records, the reference population consisted of CB animals, and BOA was ignored or considered. The x-axis represents the regression coefficient when ignoring BOA and the y-axis represents the regression coefficient when considering BOA. Panels refer to validation on CB offspring averages or individual CB records across rows, and to body weight measured at around 7 (BW7) or 35 (BW35) days across columns. Dots represent individual validation correlations of 100 replicates, and straight lines indicate x = y

For BW35, there were no clear differences in bias of GEBV between models that considered or ignored BOA. For BW7 and with validation on offspring averages, GEBV from models that considered BOA were less biased (0.55; Table 5) than those from models that ignored BOA (0.36; Table 5) in 99% of the replicates (Fig. 4, top-left). For BW7 and with validation on individual CB records, GEBV from models that considered BOA were less biased (0.67; Table 5) than those from models that ignored BOA (0.55; Table 5) in 77% of the replicates (Fig. 4, bottom-left).

## Discussion

We compared the validation correlation and bias of GEBV of PB animals for CB performance using either CB offspring averages or individual CB records as validation records. Our aim was to investigate the effect of using either a PB or CB reference population, and the effect of either ignoring or considering BOA.

It should be noted that the PB and CB animals in this study were housed in the same environment, whereas in practice, PB animals are housed in a nucleus facility and CB animals are housed in a commercial environment. As such, the estimates of $r_{pc}$ obtained here provide an upper bound for $r_{pc}$ in practical situations, where genotype-by-environment interactions may be present [27]. Consequently, the benefit of using CB information may be larger in practical situations than found here. Thus, our results on differences in validation correlations between scenarios should not be associated with the body weight traits per se, but with the value of the $r_{pc}$.

We investigated bias of GEBV by computing weighted regression coefficients of validation records on GEBV. The average coefficients across replicates were substantially lower than 1 for all scenarios, which indicates a strong bias (over-dispersion of GEBV). This bias may be due to family structure in the data and imprecision of GEBV, which may lead to a theoretical expectation of the true regression coefficient being smaller than 1 [35]. Regardless, our results show that for BW7, taking BOA into account reduced bias in almost all the replicates. For all other comparisons, differences in regression coefficients were not statistically significant because of large standard deviations of estimates across replicates To date, no other studies have evaluated the impact of considering BOA on the bias of GEBV, and therefore, it remains unclear whether considering BOA generally reduces bias or not.

### Purebred versus crossbred reference populations

As expected, our results suggest that with validation on CB offspring averages, the difference in validation correlation between using a PB and a CB reference population partly depends on the $r_{pc}$. With an $r_{pc}$ of 0.96 (BW35), the validation correlation was lower with a CB reference population than with a PB reference population, while validation correlations were similar for the CB and PB reference populations with an $r_{pc}$ of 0.80 (BW7). These results are in line with studies based on simulated [12, 13] and real data [14], thus confirming that the benefit of a CB reference population is larger for smaller values of $r_{pc}$. However, with validation on individual CB records, the validation correlation was higher with a CB reference population, regardless of the $r_{pc}$ (i.e., for both traits), which agrees with Lopes et al. [25], who analysed traits with an $r_{pc}$ of about 0.9 and also validated on CB offspring records. In addition, two other studies have shown that genotyping CB animals improves the accuracy of CB offspring GEBV using single-step genomic best linear unbiased prediction (GBLUP) [29, 36]. In the following sections, we will discuss the two validation strategies and give reasons that explain why they can result in different conclusions about the benefit of using CB information for genomic prediction.

### Validation on offspring averages

With validation on CB offspring averages, differences in validation correlations between using a PB (PB-A) versus a CB (CB-A) reference population can result from two mechanisms: (1) with an $r_{pc}$ less than 1, a CB reference population has an advantage over a PB reference population; (2) in the CB reference population, only half of the alleles originate from the sire line [12, 37], whereas all alleles originate from the sire line in the PB reference population. When the sire and dam lines are unrelated, the maternal alleles in the CB reference population introduce noise in the estimation of the sire-line genetic component because the sire-line alleles in the CB reference population explain only half of the genetic variance, whereas sire-line alleles in the PB reference population explain the full genetic variance. This results in a disadvantage for a CB reference population compared to a PB reference population. However, when the sire and dam lines are somewhat related, the dam-line allelic effects in the CB reference population may have some predictive value for the sire-line allelic effects. This would increase the accuracy of sire-line GEBV, and thus reduce the disadvantage for the CB reference population compared to the PB reference population when using a model that ignores BOA.

Observed differences in validation correlations between PB-A and CB-A depend on the balance between the aforementioned two mechanisms. To quantify the predictive value of dam-line allelic effects for sire offspring averages, we estimated sire GEBV by using only the alleles in the CB reference population that originated from the dam line. For BW7, the mean validation correlation from this model was equal to 0.03, with a standard deviation of 0.07 across replicates, whereas for BW35, the mean validation correlation was equal to 0.14 with a standard deviation of 0.07. These results indicate that the dam alleles in the CB animals may have some predictive value for sire offspring averages, which is supported by the observation that considering BOA (i.e. removing the dam alleles) decreased the validation correlation for BW35 (as discussed in later sections). For BW7, the effects of the two mechanisms resulted in similar validation correlations for PB-A and CB-A. For BW35, for which $r_{pc}$ was closer to 1, the effects of the two mechanisms resulted in a lower validation correlation with CB-A than with PB-A.

### Validation on individual offspring records

With validation on individual offspring records, differences in validation correlations between a PB reference population (PB-I) and a CB reference population (CB-I) observed in this study may be due to the same two mechanisms described above. However, the predictive value of the dam alleles is higher with validation on individual crossbred records than with validation on crossbred offspring averages of sires, for two reasons: (1) the prediction of individual CB records is partly (i.e., half) based on the dam-line alleles of those CB individuals, and (2) an individual record may have a residual genetic dam component. Thus, the CB-I validation correlations are prone to overestimate GEBV accuracies due to the contribution of dam alleles to the prediction of individual records, which contain a residual genetic dam component. For both traits (BW7 and BW35), the effects of these two mechanisms resulted in higher validation correlations with CB-I than with PB-I, but this difference was smaller for BW35 than for BW7, which was probably due to the higher $r_{pc}$ of BW35.

### Choice of validation records

As discussed in the previous sections, the difference in validation correlations of genomic predictions between using a CB and a PB reference population depend not only on the value of $r_{pc}$ but also on the choice of validation records (CB offspring averages or individual CB records). We even observed that the ranking of validation correlations with a PB versus a CB reference population changed when a different validation record was used, which raises the question of which validation record is most relevant. In practice, breeders usually aim at identifying PB selection candidates that, on average, produce the best CB offspring. Thus, the relevant validation correlation is the correlation of the GEBV of sires and their CB offspring averages. Validation on offspring averages may not be possible when the number of genotyped PB sires with phenotyped CB offspring is too small. In those cases, validation of GEBV from CB animals on their individual records may provide an alternative. However, with validation on individual records, the apparent superiority of a CB over a PB reference population will likely be inflated because, as discussed above, validation correlations from models that use a CB reference population and ignore BOA are contaminated with the predictive value of dam alleles for the residual genetic dam component in the validation records. Indeed, this inflation was reflected in a higher validation correlation with validation on individual records (0.29 for BW7 and 0.33 for BW35, [see Additional file 1: Table S1]) instead of on offspring averages (0.18 for BW7 and 0.30 for BW35, [see Additional file 1: Table S1]), when the validation correlations were compared on the same scale (i.e. scaled by the square root of the heritability and of the mean reliability, respectively). This mechanism may explain why, for traits with similar $r_{pc}$, Lopes et al. [25] found that the validation correlation was higher with a CB reference population (with validation on individual CB records), but Hidalgo et al. [14] found that the validation correlation was higher with a PB reference population (with validation on CB offspring averages). Thus, when genomic predictions using a PB versus a CB reference population are compared, validation of sire GEBV on CB offspring averages is preferred.

In the previous paragraph, we argued that, with validation on individual offspring records and when BOA is ignored, validation correlations may be inflated due to the predictive value of dam alleles. However, when BOA is considered, the dam alleles of CB animals are removed from the explanatory variables of the model and the validation correlation is not expected to be inflated. So, when validating on individual records, the benefit of using a CB reference population is better evaluated by comparing a model that uses PB information with a model that uses CB information while considering BOA. This comparison for our data showed that the CB reference population yielded a higher validation correlation than the PB reference population for BW7 (0.08 vs. 0.05) but not for BW35 (0.09 vs. 0.13). Furthermore, for this comparison, GEBV were less biased with a CB reference population than with a PB reference population for BW7 but not for BW35, although differences in regression coefficients were not statistically significant.

### Considering versus ignoring BOA

We compared the validation correlation of models that ignored (CB-A and CB-I) or considered BOA (CB-A-BOA and CB-I-BOA). With validation on offspring averages, the difference in validation correlations between considering and ignoring BOA depended on the predictive value of dam alleles in the CB animals for sire offspring averages. As shown before, this predictive value was close to zero for BW7 but larger than zero for BW35. In other words, the dam alleles introduced noise in the estimation of the genetic sire component for BW7 but this noise was less for BW35, resulting in a higher validation correlation when BOA was considered for BW7 but lower for BW35. These results suggest that taking BOA into account was beneficial for a trait with an $r_{pc}$ of 0.8 but not for a trait with an $r_{pc}$ of 0.96, which agrees with results of Sevillano et al. [26] and Lopes et al. [25], who also found that the benefit of considering BOA decreased with increasing $r_{pc}$ and heritability. It has been argued that considering BOA may improve the validation correlation when the estimated $r_{pc}$ from a model that takes BOA into account is different from a model that ignores it [38]. Our study neither confirmed nor contradicted this hypothesis because, although we observed a benefit of considering BOA for BW7, the estimate $r_{pc}$ from models that ignored or considered BOA were the same in this dataset [27].

### Implementation of BOA in practice

To our knowledge, information on BOA is currently not used in commercial crossbred evaluations. One reason may be that the algorithm to derive BOA is computationally demanding for large datasets. However, phasing algorithms are continuously being improved in terms of computational requirements [39] and computation power keeps increasing [40]. In the long term, we expect that implementation of BOA-models will depend mainly on their benefit for genomic prediction, because computing costs will be relatively small compared to other costs of a breeding program. The results of this study and those of others [25, 26] suggest that considering BOA can improve the accuracy of genomic predictions for traits with a low $r_{pc}$ and low heritability. Furthermore, as discussed above, the value of CB information for genomic prediction accuracy may be over-predicted when validation is on individual offspring records and BOA is ignored.

### Practical relevance

In this study, we investigated whether GEBV of PB animals for CB performance should be computed based on PB or CB performance measured on animals that have comparable relationships with the selection candidates. Thus, own performance records of selection candidates were ignored. In practice, however, selection candidates may have an own performance record for PB performance. For those cases, it may be more useful to compare scenarios that use only PB records with those that combine PB and CB records in a single reference population. However, some traits cannot be measured on selection candidates (e.g. carcass traits) and, as a result, GEBV can only be computed based on information from relatives. For those cases, our results provide valuable insight into the benefit of CB over PB information.

## Conclusions

Our findings show that the difference in validation correlations between using a PB or CB reference population not only depends on the $r_{pc}$ of the trait evaluated but also on the choice of the validation record. With a CB reference population, the validation correlation from validation on individual CB records can be inflated because CB offspring records contain a substantial residual genetic dam component that can be predicted by the dam alleles of CB animals. Thus, we argue that, whenever possible, validation correlations for GEBV of PB animals for CB performance should be obtained from validation on CB offspring averages, because the interest usually lies in the identification of PB animals that, on average, produce the best CB offspring. When validation on offspring averages is not possible and validation is on individual CB records, the actual benefit of using a CB reference population should be assessed by comparing the use of a PB reference population with the use of a CB reference population with BOA considered. For this comparison, our results show that the validation correlation with a CB reference population was equal to or higher than with a PB reference population for a trait with an $r_{pc}$ of 0.8 but lower for a trait with an $r_{pc}$ of 0.96. In addition, in our population, taking BOA into account was beneficial for a trait with an $r_{pc}$ of 0.8 but not for a trait with an $r_{pc}$ of 0.96.

## Additional file

**Additional file 1: Table S1.** Scaled^a^ mean validation correlations for BW7 and BW35.

## Data Availability

The data used in the present study were provided by Cobb-Vantress, Inc and are not publicly accessible. Raw phenotypes and genotypes are only available through Cobb-Vantress.

## References

[1] Wei M, van der Werf JH (1995). Genetic correlation and heritabilities for purebred and crossbred performance in poultry egg production traits. J Anim Sci.

[2] Lukaszewicz M, Davis R, Bertrand JK, Misztal I, Tsuruta S (2015). Correlations between purebred and crossbred body weight traits in Limousin and Limousin-Angus populations. J Anim Sci.

[3] Wientjes YCJ, Calus MPL (2017). BOARD INVITED REVIEW: the purebred-crossbred correlation in pigs: A review of theory, estimates, and implications. J Anim Sci.

[4] Dekkers JC (2007). Marker-assisted selection for commercial crossbred performance. J Anim Sci.

[5] Lutaaya E, Misztal I, Mabry JW, Short T, Timm HH, Holzbauer R (2001). Genetic parameter estimates from joint evaluation of purebreds and crossbreds in swine using the crossbred model. J Anim Sci.

[6] Wei M, van der Steen HAM, van der Werf JHJ, Brascamp EW (1991). Relationship between purebred and crossbred parameters. J Anim Breed Genet.

[7] Zumbach B, Misztal I, Tsuruta S, Holl J, Herring W, Long T (2007). Genetic correlations between two strains of Durocs and crossbreds from differing production environments for slaughter traits. J Anim Sci.

[8] Lo LL, Fernando RL, Grossman M (1997). Genetic evaluation by BLUP in two-breed terminal crossbreeding systems under dominance. J Anim Sci.

[9] VanRaden PM (2008). Efficient methods to compute genomic predictions. J Dairy Sci.

[10] Meuwissen THE, Hayes BJ, Goddard ME (2001). Prediction of total genetic value using genome-wide dense marker maps. Genetics.

[11] Hayes BJ, Visscher PM, Goddard ME (2009). Increased accuracy of artificial selection by using the realized relationship matrix. Genet Res (Camb).

[12] Van Grevenhof IE, Van Der Werf JH (2015). Design of reference populations for genomic selection in crossbreeding programs. Genet Sel Evol.

[13] Esfandyari H, Sorensen AC, Bijma P (2015). A crossbred reference population can improve the response to genomic selection for crossbred performance. Genet Sel Evol.

[14] Hidalgo AM, Bastiaansen JW, Lopes MS, Calus MP, de Koning DJ (2016). Accuracy of genomic prediction of purebreds for cross bred performance in pigs. J Anim Breed Genet.

[15] de Roos AP, Hayes BJ, Spelman RJ, Goddard ME (2008). Linkage disequilibrium and persistence of phase in Holstein-Friesian, Jersey and Angus cattle. Genetics.

[16] Veroneze R, Bastiaansen JW, Knol EF, Guimaraes SE, Silva FF, Harlizius B (2014). Linkage disequilibrium patterns and persistence of phase in purebred and crossbred pig (Sus scrofa) populations. BMC Genet.

[17] Pengelly RJ, Gheyas AA, Kuo R, Mossotto E, Seaby EG, Burt DW (2016). Commercial chicken breeds exhibit highly divergent patterns of linkage disequilibrium. Heredity (Edinb).

[18] Fu W, Dekkers JC, Lee WR, Abasht B (2015). Linkage disequilibrium in crossbred and pure line chickens. Genet Sel Evol.

[19] Wientjes YCJ, Veerkamp RF, Bijma P, Bovenhuis H, Schrooten C, Calus MPL (2015). Empirical and deterministic accuracies of across-population genomic prediction. Genet Sel Evol.

[20] Fisher RA (1918). The correlation between relatives on the supposition of mendelian inheritance. Philos Trans R Soc Edinb.

[21] Falconer DS (1952). The problem of environment and selection. Am Nat.

[22] Vandenplas J, Calus MPL, Sevillano CA, Windig JJ, Bastiaansen JWM (2016). Assigning breed origin to alleles in crossbred animals. Genet Sel Evol.

[23] Ibañez-Escriche N, Fernando RL, Toosi A, Dekkers JC (2009). Genomic selection of purebreds for crossbred performance. Genet Sel Evol.

[24] Christensen OF, Madsen P, Nielsen B, Su G (2014). Genomic evaluation of both purebred and crossbred performances. Genet Sel Evol.

[25] Lopes MS, Bovenhuis H, Hidalgo AM, van Arendonk JAM, Knol EF, Bastiaansen JWM (2017). Genomic selection for crossbred performance accounting for breed-specific effects. Genet Sel Evol.

[26] Sevillano CA, Vandenplas J, Bastiaansen JWM, Bergsma R, Calus MPL (2017). Genomic evaluation for a three-way crossbreeding system considering breed-of-origin of alleles. Genet Sel Evol.

[27] Duenk P, Calus MPL, Wientjes YCJ, Breen VP, Henshall JM, Hawken R (2019). Estimating the purebred-crossbred genetic correlation of body weight in broiler chickens with pedigree or genomic relationships. Genet Sel Evol.

[28] Calus MPL, Vandenplas J, Hulsegge B, Borg R, Henshall JM, Hawken R. Derivation of parentage and breed-origin of alleles in a crossbred broiler dataset. In: Proceedings of the 11th world congress on genetics applied to livestock production: 11–16 Feb 2018; Auckland; 2018.

[29] Xiang T, Nielsen B, Su G, Legarra A, Christensen OF (2016). Application of single-step genomic evaluation for crossbred performance in pig. J Anim Sci.

[30] Sargolzaei M, Chesnais JP, Schenkel FS (2014). A new approach for efficient genotype imputation using information from relatives. BMC Genomics.

[31] Sevillano CA, Vandenplas J, Bastiaansen JW, Calus MP (2016). Empirical determination of breed-of-origin of alleles in three-breed cross pigs. Genet Sel Evol.

[32] Hickey JM, Kinghorn BP, Tier B, Wilson JF, Dunstan N, van der Werf JHJ (2011). A combined long-range phasing and long haplotype imputation method to impute phase for SNP genotypes. Genet Sel Evol.

[33] Wientjes YCJ, Bijma P, Vandenplas J, Calus MPL (2017). Multi-population genomic relationships for estimating current genetic variances within and genetic correlations between populations. Genetics.

[34] Cameron ND (1997). Selection indices and prediction of genetic merit in animal breeding.

[35] Legarra A, Reverter A (2018). Semi-parametric estimates of population accuracy and bias of predictions of breeding values and future phenotypes using the LR method. Genet Sel Evol.

[36] Lourenco DAL, Tsuruta S, Fragomeni BO, Chen CY, Herring WO, Misztal I (2016). Crossbreed evaluations in single-step genomic best linear unbiased predictor using adjusted realized relationship matrices. J Anim Sci.

[37] Moghaddar N, Swan AA, van der Werf JHJ (2014). Comparing genomic prediction accuracy from purebred, crossbred and combined purebred and crossbred reference populations in sheep. Genet Sel Evol.

[38] Sevillano CA. Genomic evaluation considering the mosaic genome of the crossbred pig. PhD thesis, Wageningen University. 2018.

[39] Loh P-R, Danecek P, Palamara PF, Fuchsberger C, Reshef YA, Finucane KH (2016). Reference-based phasing using the Haplotype Reference Consortium panel. Nat Genet.

[40] Denning PJ, Lewis TG (2016). Exponential laws of computing growth. Commun ACM.

