# Correction to: Validation of genomic predictions for body weight in broilers using crossbred information and considering breed-of-origin of alleles

**DOI:** 10.1186/s12711-019-0507-1

**Published:** 2019-11-11

**Authors:** Pascal Duenk, Mario P. L. Calus, Yvonne C. J. Wientjes, Vivian P. Breen, John M. Henshall, Rachel Hawken, Piter Bijma

**Affiliations:** 1 Animal Breeding and Genomics, Wageningen University and Research, P.O. Box 338, 6700 AH Wageningen, The Netherlands; 2 Cobb-vantress Inc., Siloam Springs, AR 72761-1030 USA

## Correction to: Genet Sel Evol (2019) 51:38 10.1186/s12711-019-0481-7

Following publication of original article [1], we noticed that there was an error: Eq. (3) on page 5 is the genomic relationship matrix that *ignores* the BOA (${\mathbf{G}}$), whereas it should be the genomic relationship matrix that *considers* the BOA (${\mathbf{G}}_{BOA}$). Hence, Eq. (3) should be:

3 $$ {\mathbf{G}}_{BOA} = \left[ {\begin{array}{*{20}c} {{\mathbf{G}}_{{BOA, {\text{PB}}}} } & {{\mathbf{G}}_{{BOA,{\text{PB}} - {\text{CB}}}} } \\ {{\mathbf{G}}_{{BOA,   {\text{CB}} - {\text{PB}}}} } & {{\mathbf{G}}_{{BOA, {\text{CB}}}} } \\ \end{array} } \right] = \left[ {\begin{array}{*{20}c} {\frac{{{\mathbf{M}}_{\text{PB}} {\mathbf{M}}_{\text{PB}}^{{'}} }}{{\sum 2p_{j} \left( {1 - p_{j} } \right)}}} & {\frac{{{\mathbf{M}}_{\text{PB}} {\mathbf{T}}_{\text{CB}}^{{'}} }}{{\sum 2p_{j} \left( {1 - p_{j} } \right)}}} \\ {\frac{{{\mathbf{T}}_{\text{CB}} {\mathbf{M}}_{\text{PB}}^{{'}} }}{{\sum 2p_{j} \left( {1 - p_{j} } \right)}}} & {\frac{{{\mathbf{T}}_{\text{CB}} {\mathbf{T}}_{\text{CB}}^{{'}} }}{{\sum 2p_{j} \left( {1 - p_{j} } \right)}}} \\ \end{array} } \right]. $$

## References

[1] Duenk P, Calus MPL, Wientjes YCJ, Breen VP, Henshall JM, Hawken R, Bijma P (2019). Validation of genomic predictions for body weight in broilers using crossbred information and considering breed-of-origin of alleles. Genet Sel Evol..

