# Supplementary material for: Validation of genomic predictions for body weight in broilers using crossbred information and considering breed-of-origin of alleles
Source: Genet Sel Evol. 2019 Jul 8;51:38. doi: 10.1186/s12711-019-0481-7 (PMC6613268; doi:10.1186/s12711-019-0481-7)
Supplement: Supplementary file 1 — Additional file 1: Table S1. Scaleda mean validation correlations for BW7 and BW35. [file 12711_2019_481_MOESM1_ESM.docx]

Table S1. Scaled^a^ mean validation correlations for BW7 and BW35

| **Scenario** | **Reference** | **Validation** | **BW7** | | **BW35** | |
| --- | --- | --- | --- | --- | --- | --- |
|  |  |  | **Mean^b^** | **sd^c^** | **Mean^b^** | **sd^c^** |
| PB-A | PB | Offspring averages | 0.18 | 0.037 | *0.41* | 0.036 |
| CB-A | CB | Offspring averages | 0.18 | 0.067 | 0.30 | 0.069 |
| CB-A-BOA | CB | Offspring averages | *0.23* | 0.066 | 0.25 | 0.067 |
|  |  |  |  |  |  |  |
| PB-I | PB | Individual records | 0.11 | 0.032 | 0.28 | 0.028 |
| CB-I | CB | Individual records | *0.29* | 0.044 | *0.33* | 0.042 |
| CB-I-BOA | CB | Individual records | 0.17 | 0.056 | 0.18 | 0.051 |

^a^Scaled correlations were computed as the unscaled validation correlation divided by the square-root of the heritability (for validation on individual records), or divided by the square-root of the weighted mean reliability (for validation on offspring averages). Across scenarios, the average weighted mean reliability was 0.77 for both BW7 and BW35.

^b^Reported values are means of 100 replicates. Highest mean validation correlations per validation record and per trait are in italics

^c^Reported values are standard deviations of validation correlations of 100 replicates.
